# Evidence for the coupling of refill liquids content and new particle formation in electronic cigarette vapors

**DOI:** 10.1038/s41598-022-21798-w

**Published:** 2022-11-03

**Authors:** Oluwabunmi Dada, Karina Castillo, Miranda Hogan, Marie-Cecile G. Chalbot, Ilias G. Kavouras

**Affiliations:** 1 Department of Environmental Health Sciences, University of Alabama at Birmingham, Birmingham, AL 35219 USA; 2 Department of Environmental, Occupational and Geospatial Health Sciences, CUNY Graduate School of Public Health and Health Policy, New York, NY 10025 USA; 3 Department of Occupational Safety, Murray State University, Murray, KY 42071 USA; 4 Department of Biological Sciences, CUNY College of Technology, Brooklyn, NY 11201 USA

**Keywords:** Risk factors, Chemical engineering, Atmospheric chemistry

## Abstract

The size and chemical content of particles in electronic cigarette vapors (e-vapors) dictate their fate in the human body. Understanding how particles in e-vapors are formed and their size is critical to identifying and mitigating the adverse consequences of vaping. Thermal decomposition and reactions of the refill liquid (e-liquid) components play a key role in new particles formation. Here we report the evolution of particle number concentration in e-vapors over time for variable mixtures of refill e-liquids and operating conditions. Particle with aerodynamic diameter < 300 nm accounted for up to 17% (or 780 μg/m^3^) of e-vapors particles. Two events of increasing particle number concentration were observed, 2–3 s after puff completion and a second 4–5 s later. The intensity of each event varied by the abundance of propylene glycol, glycerol, and flavorings in e-liquids. Propylene glycol and glycerol were associated with the first event. Flavorings containing aromatic and aliphatic unsaturated functional groups were strongly associated with the second event and to a lesser extent with the first one. The results indicate that particles in e-vapors may be formed through the heteromolecular condensation of propylene glycol, glycerol, and flavorings, including both parent chemicals and/or their thermal decomposition products.

Electronic cigarettes (e-cigs) use is increasing among smokers who want to quit or reduce smoking and inadvertently, by adolescence, youth and adults who perceive the device to be safer than tobacco smoking due to pervasive advertising by e-cig manufacturers^1,2^. Yet, the short- and long-term effects of e-cigs are largely unknown and poorly understood^3^. Outbreaks of e-cigarette or vaping use-associated lung injury (EVALI) in adults and other respiratory related illnesses and deaths were reported in the US^4,5^.

E-cigs are composed of: (i) a cartridge that accommodates the nicotine solution (refill e-liquid); (ii) a heating element (atomizer) that vaporizes the e-liquid; and (iii) battery assembly including microprocessor and mouthpiece^6,7^. Refill e-liquid solutions contain nicotine, the primary addictive chemical, solvents (propylene glycol (PG) and glycerol (G)), and chemicals species used as flavorings and preservatives^7^. The design, size, and operating features have changed over the past five years; however, the underlying operating principle, i.e., delivery of nicotine through heating, is the same. Newer devices are easier to operate and customize individual components and conditions yielding a great variety of products and experiences. They also add substantially to the already large range of exposure scenarios to e-vapors due to e-liquids flavors, heating temperature, the vapor pressure of chemicals, and user behavior^7–9^. This directly impacts the precision, robustness, and reproducibility of in vitro, in vivo, and human studies to understand the biological and toxicological responses to e-vapors.

The particle number, mass, and size distribution of e-vapors compared to tobacco smoke were previously studied^10–13^. The count median diameter in tobacco smoke was higher than those in e-vapors, indicating that much smaller particles, penetrating deeper in the alveolar region, and translocating easier to the bloodstream, are produced by e-cigs^10^. The particle number concentration (PNC) did not vary substantially for e-vapors with different nicotine levels^12^. PNC was measured in mainstream e-vapors compared to those measured for mainstream tobacco smoke and secondhand e-vapors following had a bimodal distribution with maxima at 30 nm and 90 nm^13^. Fuoco et al.^14^ also reported that e-cig devices and products had higher PNC than tobacco smoke, with the number of puffs made and nicotine concentration in e-liquids being important determinants of PNC generated in e-cigs. Zhao et al.^15^ showed that e-vapors were comprised of particles in the order of 10^6^–10^7^ part/cm^3^ and a mode diameter of about 200 nm depending on e-cigs devices, flavors, puff protocols, and voltage. A multi-mode particle size distribution with maxima at aerodynamic diameter (d_a_) of 40, 200, and 1000 nm were reported for e-vapors at different voltage settings^16^. Undiluted e-vapors had PNC in the span of 10^9^ particles/cm^3^ and particle diameter in the range of 250–450 nm, while for diluted e-vapors, the particle diameter decreased 50 nm with lower PNC^17^. The particle concentration of e-cigs was 300–3000 times higher than PNC levels in ambient air^18^. The particle formation mechanism and the role of e-liquids contents are poorly understood. Previous studies have demonstrated that aldehydes in e-vapors were not present in e-liquids but were formed from propylene glycol and glycerol oxidation reactions during heating^19–21^. Thermal decomposition and oxidation chemical species may also be formed by flavorings in e-liquids^22^.

The objective of this study is to determine the linkage between e-vapors PNC and e-liquids chemical composition to better understand the particle formation mechanisms, including the potential for secondary aerosol formation. E-vapors were generated by commercially available devices using a set of custom-made reference solutions and commercially available e-liquids. The chemical content of e-liquids was previously determined by nuclear magnetic resonance (NMR) spectroscopy^23^.

## Results

### Effect of flavor and operational power on particle mass size distribution

The experimental conditions and total e-vapor particle mass are depicted in Table 1. There was no difference on particle mass of menthol (4.5 ± 1.0 mg/m^3^) and tobacco-flavored (5.8 ± 0.2 mg/m^3^) e-vapors generated at 180 W (*p* = 0.028). An increase of nicotine content led to an increase of particle mass from 2.1 ± 0.3 to 4.5 ± 1.0 mg/m^3^; however, high particle mass concentrations were also measured for nicotine-free e-vapors (3.9 ± 0.5 mg/m^3^) (*p* = 0.133). Particle mass increased significantly as the operational voltage increased from 120 to 180 W (*p* < 0.001). The trend is not linear for lower wattage and within the recoomended operating voltage by the manufacturer. The high particle mass at 90 W maybe associated with larger particles (MMAD 1.26 ± 0.08 μm) as compared to those generated at 150 W (particle mass = 3.5 ± 0.6 mg/m^3^ and MMAD of 0.78 ± 0.02 μm).

**Table 1:** Particle mass and size distribution data

Flavor (nicotine conc.)	Power (W)	Particle mass conc. (mg/m^3^)	% PM_<0.3_^a^	MMAD (μm)
Menthol^b^ (0 mg/ml)	180	3.9 ± 0.5	15	0.97 ± 0.04
Menthol^b^ (12 mg/ml)	180	2.1 ± 0.3	11	1.04 ± 0.05
Menthol^b^ (24 mg/ml)	180	4.5 ± 1.0	11	1.04 ± 0.05
Tobacco^c^ (24 mg/ml)	90	3.2 ± 0.5	13	1.26 ± 0.08
Tobacco^c^ (24 mg/ml)	120	0.9 ± 0.1	14	1.98 ± 0.09
Tobacco^c^ (24 mg/ml)	150	3.5 ± 0.6	17	0.78 ± 0.02
Tobacco^c^ (24 mg/ml)	180	5.8 ± 0.2	12	0.97 ± 0.02

± corresponds to the standard error of the mean (n = 16) mg/ml milligrams per milliliter of e-liquid, W Watt, mg/m^3^ milligrams per cubic meter of air, PM_<0.3_ particles with aerodynamic diameter less than 0.3 μm, MMAD mass median aerodynamic diameter, μm micrometer.

^a^calculated using the mass of e-vapors collected on impactor stages,

^b^ANOVA with Tukey HSD *p* = 0.133

^c^ANOVA with Tukey HSD *p* < 0.001.

**Figure 1:**
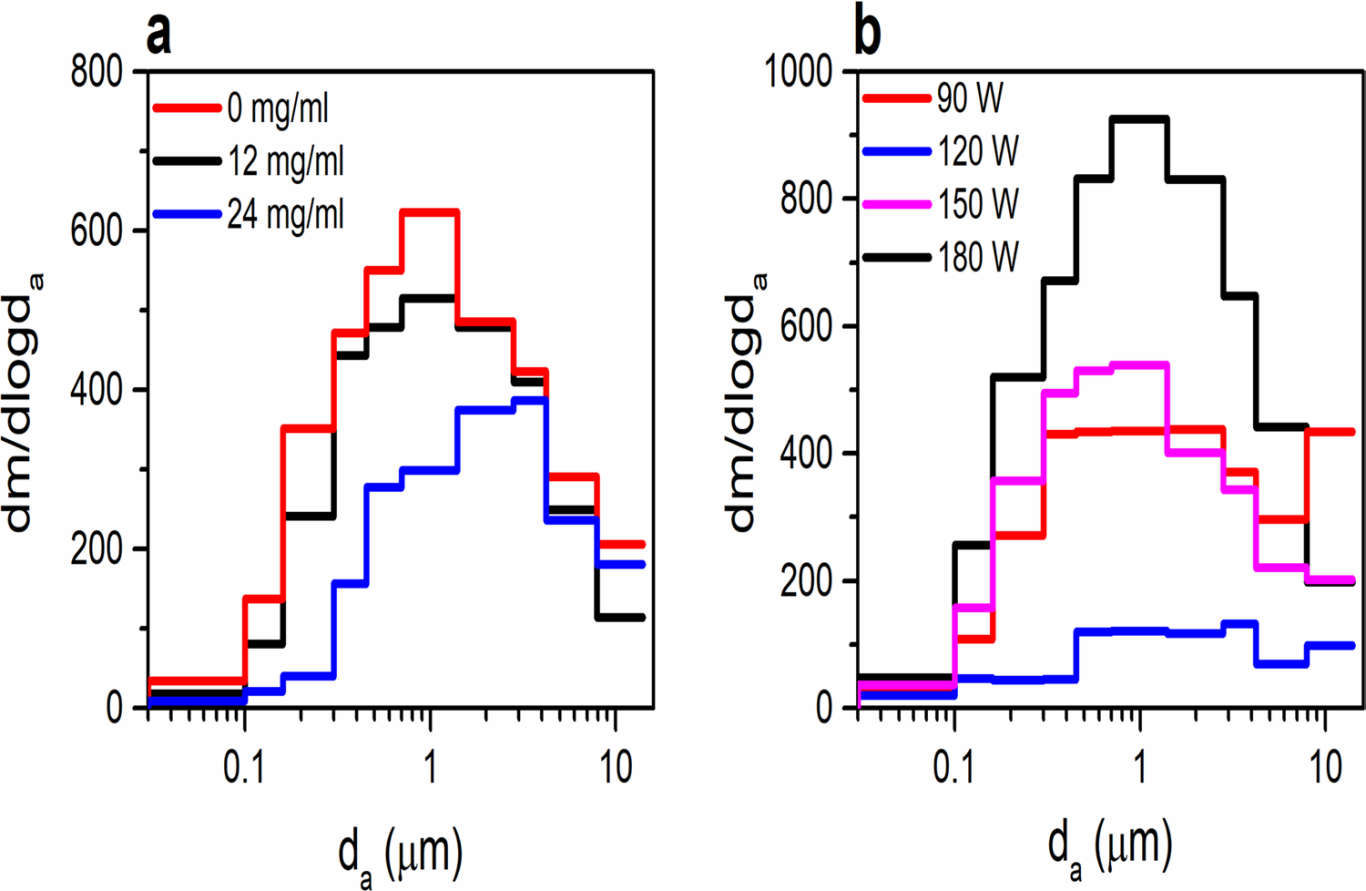
Normalized particle mass size distribution of e-vapors from (**a**) menthol flavored e-liquids of 0, 12, and 24 mg/ml nicotine concentrations (at 180 W) and (**b**) tobacco flavored e-liquids at 90, 120, 150 and 180 W (24 mg/ml nicotine content) using the 10-stage real time QCM cascade impactor. Experiments were carried out using a 2 s puff and 15 s wait time (n = 16). QCM Quartz crystal microbalance, mg/mL milligrams per milliliter, μm micrometer, W watts.

Figure 1 shows the size distribution of e-vapors generated for (a) variable nicotine contents and (c) variable operational voltages. A one-mode distribution with maxima for particles with 0.7 < d_a_ < 1.4 µm was observed for most of e-vapors. This corresponded to median mass aerodynamic diameter (MMADs) from 0.78 ± 0.01 μm to 1.04 ± 0.05 μm. For e-vapors generated at 90 W and 120 W, a second maxima ad particles with 7.9 < d_p_ < 14 µm was observed corresponding to MMADs of 1.26 ± 0.08 μm and 1.98 ± 0.09 μm. For all experiments, particles with d_a_ < 300 nm accounted for up to 17% of total particle mass (i.e., from 135 to 780 μg/m^3^ by mass).

### Ultrafine particle formation by e-vapors major components

**Figure 2:**
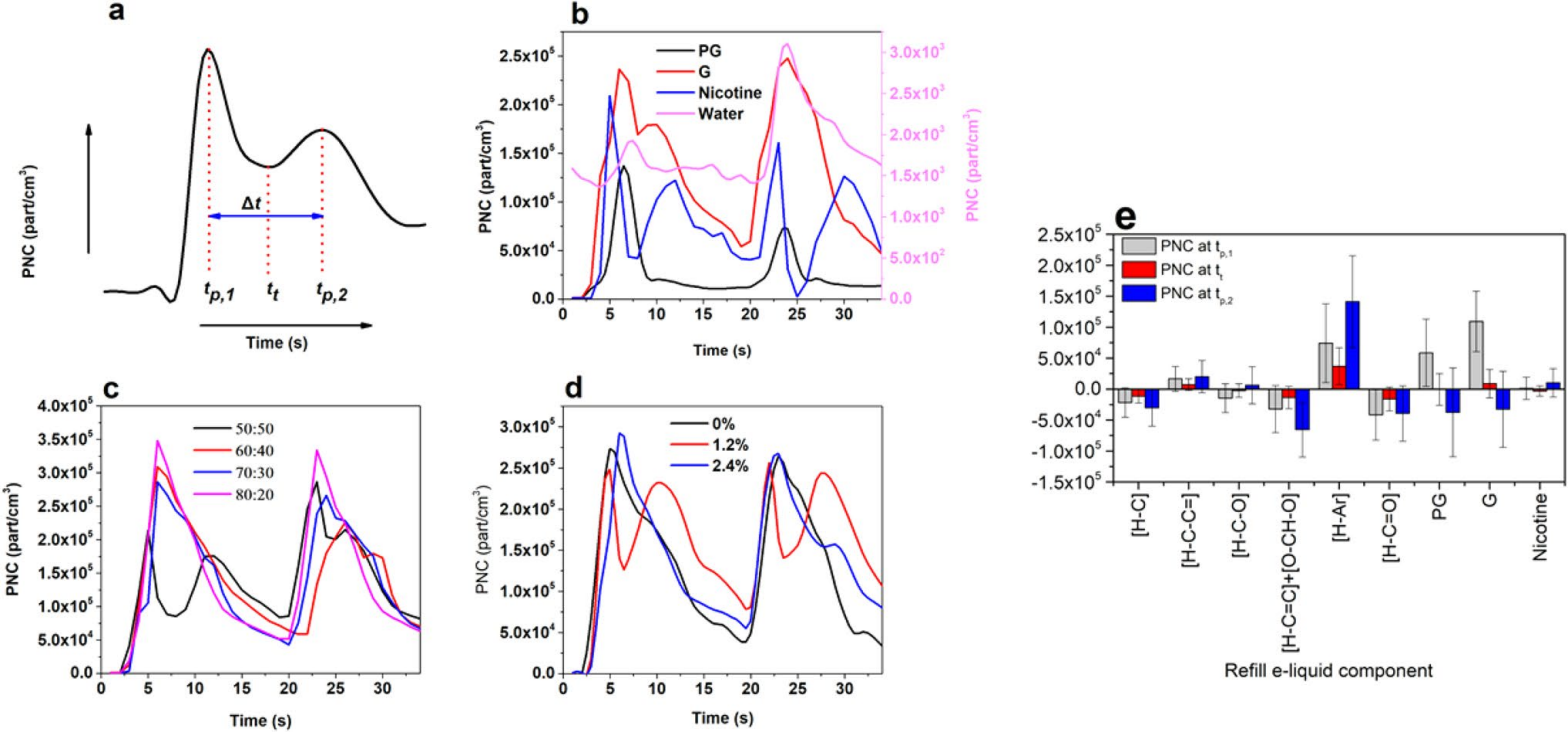
(**a**) Schematic representation of PNC over time and characteristics time points (first PNC peak at t_p,1_; second PNC peak at t_p,2_, time difference between two PNC peaks (Δt) and time of the local minimum (trough, t_t_) between two peaks; PNC for two consecutive puffs for (**b**) reference solutions of PG, G, water, nicotine, (**c**) nicotine and flavorings-free mixtures of PG: G (50:50, 60:40, 70:30 and 80:20 by volume), (**d**) commercial flavored e-liquids with variable nicotine content^a^, and (**e**) contribution of refill e-liquid components^a^ on PNC at t_p,1_, t_t_ and t_p,2_. Experiments were carried out using a 2 s puff and 15 s wait time at 100 W (n = 16). ^a^refill e-liquid composition was characterized using proton nuclear magnetic resonance (^1^H-NMR) spectroscopy^23^. part/cm^3^ particles per cubic centimeter, s micrometer, PG propylene glycol, G glycerol, [H–C] saturated aliphatic, [H–C–C=] unsaturated aliphatic, [H–C–O] saturated oxygenated, [H–C=C] vinylic, [O–CH–O] acetalic, [H–Ar] aromatic, [H–C=O] carbonyl non-exchangeable organic hydrogen.

Figure 2 shows (a) a schematic representation of PNC concentration over time in e-vapors and the characteristic timepoints, and PNC of e-vapors generated from (b) pure PG, G, water, and nicotine, the predominant components of e-liquids, (c) nicotine-free PG:G mixtures, and (d) commercially available e-liquids with variable nicotine content. The contributions of refill e-liquid contents on PNC at the three characteristic t_p,1_ (time of the first PNC maxima following the completion of a puff), t_p,2_ (time of the second PNC maxima following the completion of a puff) and t_t_ (time of the local minimum between the first and second PNC timepoints) are shown in Fig. 2e.

Rapid PNC increase was observed 2 s after puff completion, reaching its first maximum (and global maximum, t_p,1_) within 2.7 s for nicotine and water, 3.3 s for PG, and 4.3 s for G (Fig. 2b). PNC declined rapidly, reaching a local minimum within a couple of seconds (t_t_) followed by a second PNC increase (at t_p,2_) within 3.7 to 4.8 s after the first peak (Δt) (Fig. 2b). More particles were generated by G (68,768 ± 10,798 part/cm^3^), followed by nicotine (52,288 ± 8524 part/cm^3^) and PG (48,411 ± 10,901 part/cm^3^). The lowest PNC was measured for water (3092 ± 443 part/cm^3^). For nicotine- and flavoring-free PG:G mixtures, the second PNC peak phased out as the abundance of propylene glycol increased (Fig. 2c). The timing of the first mode was comparable for all reference PG:G mixtures. The addition of water (10%) slightly decreased the mean PNC. On the other hand, PNC increased for solutions containing 0.6% nicotine compared to nicotine-free reference solutions but slightly decreased for solutions with a higher abundance of nicotine (1% and 2.5%). The pattern for nicotine-free vanilla flavored e-vapors was comparable to that observed for reference PG:G solutions. E-vapors with nicotine yielded a clear bimodal pattern which was consistent with that previously observed for nicotine alone (Fig. 2b,d).

### Ultrafine particle formation by commercially available e-liquid refill solutions

Table 2 shows the mean PNC, t_p,1,_ and Δt for e-vapors generated with commercially available e-liquids clustered based on the PG:G ratio and flavorings molar ratio^23^. For the flavorings molar ratio, the ranges represented the 20th, 40th, 60th, and 80th percentile to obtain comparable number of samples for each range. PNC in flavored e-vapors declined from 123,151 ± 15,127 part/cm^3^ to 87,684 ± 6,104 part/cm^3^ as PG abundance increased. PNC increased from 97,327 ± 8721 part/cm^3^ to 123,069 ± 20,409 part/cm^3^ as the flavorings molar ratio increased up to 0.035 (or 3.5%), followed by a sharp decline 99,755 ± 7,766 part/cm^3^ for e-vapors with higher flavorings molar ratios. There was no significant change on t_p,1_ and t_p,2_. Negative in Fig. 2e, indicated that chemical species may enhance particle growth and/or accumulation, thus fewer and larger particles are formed. PG and G appeared to favor new particles at t_p,1_, and particle growth at t_p,2_. The highest contribution to PNC for all timepoints was computed for [H–Ar] and [H–C–C=] functionalities of flavorings. Flavorings containing [H–C], [H–C–O], [H–C=C], [O–CH–O], and [H–C–O] groups seem to enhance particle condensation and growth.

**Table 2:** Summary of PNC concentrations and events in e-vapors. ± standard error of the mean.

Refill e-liquid solutions^a^	PNC (part/cm^3^)	t_p,1_ (s)	Δt (s)
PG:G 30–35	123,151 ± 15,127	2.6 ± 0.2	5.1 ± 0.3
PG:G 40–45	99,372 ± 5153	2.9 ± 0.2	4.3 ± 0.6
PG:G 50–55	100,255 ± 7537	2.4 ± 0.1	5.5 ± 0.2
PG:G 60–65	87,684 ± 6104	2.7 ± 0.5	4.6 ± 0.4
FMR^b^ < 0.022	97,327 ± 8721	2.6 ± 0.3	4.8 ± 0.4
0.022 < FMR < 0.030	101,847 ± 10,419	2.5 ± 0.1	5.5 ± 0.3
0.030 < FMR < 0.035	123,069 ± 20,409	2.3 ± 0.2	5.5 ± 0.4
0.035 < FMR < 0.049	98,550 ± 2857	2.8 ± 0.2	3.5 ± 0.9
FMR > 0.049	99,755 ± 7766	3.3 ± 0.2	4.8 ± 0.4

Experiments were carried out using a 2 s puff and 15 s wait time at 100 W (n = 16). PNC particle number concentration, part/cm^3^ particles per cubic centimeter, t_p,1_ time of the first peak PNC, s seconds, Δt time between two consecutive PNC, PG, propylene glycol, G glycerol, FMR flavorings molar ratio.

^a^Refill e-liquid composition was characterized using proton nuclear magnetic resonance (^1^H-NMR) spectroscopy^23^,

^b^Computed as the ratio of the total non-exchangeable organic hydrogen molar concentration to the total molar concentration of propylene glycol, glycerol, and nicotine as determined by ^1^H-NMR spectrsocopy^23^.

## Discussion

We observed particle formation during vaping for both e-liquid chemical species in pure form, mixtures, and commercially available e-liquids. The heating temperature of the coil was 198.75 °C, lower than the boiling temperatures of nicotine (277.875 °C), PG (211.75 °C), and G (326.25 °C), but sufficient to trigger their evaporation. It exceeds water’s boiling temperature (100 °C); therefore, the generated e-vapors could contain significant quantities of water vapors. As a result, evaporated solvents may be present as gases and vapors^16^. E-vapors may rapidly undergo thermal expansion and cooling to ambient temperatures. In a user, inhaled e-vapors may cool to 37 °C. The cooling may result in the partitioning of gaseous species in the vapor/aerosol phase yielding the formation of particles by condensation^14^. Concurrently, chemical reactions of gases may occur rapidly including the production of hydroxyl radicals that rapidly react with unsaturated and oxygenated compounds to form low volatility carbonyl or carboxyl species that can partition to the aerosol phase^24–26^.

In this study, we observed a multimodal distribution of PNC over time in e-vapors in reference mixtures and e-liquids. This may be indicative of the synergies among chemicals in altering the supersaturation ratio and enhancing molecular bond interactions (e.g., Van der Waals or hydrogen bonds) to enhance homogeneous homo-/hetero-molecular condensation^27^. Furthermore, the presence of pre-existing vapors may facilitate particle formation through heterogeneous homo-/hetero-molecular condensation^28^. For propylene glycol, glycerol and nicotine, their vapor pressure may increase by a factor of 6500 to 10,000 for the heating temperature of 198.78 °C (calculated using the Antoine equation) compared to the vapor pressure of the chemicals at 25 °C^29,30^. As the vapors cool down, the supersaturation ratio would be substantially higher than 1 (up to 5000 for an ambient temperature of 37 °C). This is sufficient to trigger the formation of new particles immediately through homogeneous homo- or heteromolecular condensation. The heteromolecular condensation between propylene glycol and glycerol may be favored due to the formation of polymers through hydrogen bonds. This mechanism may be responsible for the rapid PNC increase at t_p,1_. The mode was relatively short-lived because of continuous growth and accumulation of particles yielding larger particles and lower PNC^31^. The sharpness of the decline may be associated with the chemical content of e-vapor. For example, chemical species with lower vapor pressure would facilitate a prolonged accumulation process and slower decline in particle number concentration. The vapor pressures of nicotine (5.0 × 10^–5^ atm), propylene glycol (1.6 × 10^–4^ atm) may justify the relatively sharp declines in propylene glycol and nicotine particle number concentrations as compared to those observed for glycerol (2.2 × 10^–7^ atm)^32^.

The second PNC peak may be indicative of the formation of new secondary particles even though the concentrations of the newly formed chemical compounds may be below the saturation concentration, but supersaturation conditions may exist due to the presence of other gases and pre-existing particles^33,34^. For propylene glycol, nicotine and glycerol, the first-rate reaction constant at 25 °C were equal to 8.38 × 10^–11^ cm^3^/(molecule s), 21.5 × 10^–12^ cm^3^/(molecule s) and 1.5 × 10^23^ cm^3^/(molecule s)^35–37^. Assuming a molar concentration of nicotine, propylene glycol and glycerol of 1.23 × 10^15^, 4.18 × 10^15^ and 5.44 × 10^12^ molecules/cm^3^ (in equilibrium between gas and vapor phase based on compound’s vapor pressure at 1 atm and 25 °C using the ideal gas law), the estimated half-life time would be 1/100 of a millisecond for nicotine and lower for propylene glycol and glycerol (assuming first order kinetics and half-life t_1/2_ = 1/k[A]). The oxidation of e-liquid components has been suggested as the primary mechanism for formaldehyde, nitrosamines, and other chemical species in e-vapors^38,39^. This mechanism is also possible for flavorings, particularly for chemicals with unsaturated and aromatic structures and carbonyl groups. For propylene glycol and glycerol, reactions with OH radicals involve the abstraction of a proton from a hydroxyl group to form an alkoxy radical. On the other hand, in compounds with *sp*^*2*^-hybridized carbon, the hydroxyl radicals proceed through the addition of the hydroxyl group in the double bond^40^. The step of addition requires less energy than the step of abstraction resulting in faster reaction rate kinetics for alkenes and aromatics as compared to alcohols. This is consistent with the stronger association of aliphatic unsaturated and aromatic functional groups with particle number concentrations for the second peak. Given the abundance of their precursors (propylene glycol, glycerol, and flavorings) and the pre-existing particles formed from the cooling of hot vapors, the secondary products may form secondary particles by homogeneous or heterogeneous nucleation.

In pure form or reference solutions, propylene glycol, glycerol, water, and nicotine were the only chemical species for the formation of particles, dictating the interactions between them and, therefore, the possibility to overestimate the abundance of a chemical in the generated particle number concentration. In e-liquids, other chemicals are also present that may also interact with glycerol, propylene glycol, and nicotine. We have previously identified menthol, vanillin, and benzaldehyde in commercially available liquids used in this study^23^. Their vapor pressures range from 1.5 × 10^–7^ atm for vanillin to 1.0 × 10^–5^ atm for menthol and 1.6 × 10^–3^ atm for benzaldehyde^41^. These chemical species also have the potential to produce particles through homogeneous nucleation, as described previously. Assuming equal quantities of nicotine and benzaldehyde in refill e-liquids, benzaldehyde molarity in e-vapors would be two times higher than nicotine molarity. Moreover, these chemical species (and others identified as flavorings in e-liquids) contain functional groups that can form dimers or trimers with propylene glycol and glycerol^42^. This could explain the reduced association of propylene glycol with particle number concentration in commercial e-liquids compared to those in the reference solutions.

The formation of particles through condensation of hot vapors (i.e. primary) and low vapor pressure products (i.e. secondary) is consistent with previous studies showing multimode size distributions of e-vapors^13,15–17^ at particle sizes that have been associated with secondary organic aerosol formation in atmospheric conditions^33^. Both processes have been shown to generate particles in the ultrafine range. However, the chemical content of primary and secondary particles may be different, affecting the long-term health impacts of e-vapors.

Secondary particles may contain toxic and carcinogenic chemicals in low concentrations, sufficient to trigger particle formation in a synergistic manner and long-term health outcomes^43–45^. The rapid formation of primary and secondary particles and the inherent variability due to inconsistencies in e-liquid composition and user patterns pose significant limitations in personal exposure and epidemiological investigations. In controlled experiments, continuous particle size distribution measurements in the ultrafine size range may not differentiate between the two processes because of the sequential particle classification and detection that requires measurement time longer than the observed timescale of particle formation in this study. The relative abundance and chemical content of primary and secondary particles may be assessed with concurrent collection and chemical analysis of size fractionated samples.

In conclusion, e-cigarette users may be exposed to unsafe and potentially unhealthy levels of particulate matter in flavored e-vapors, including tobacco-flavored e-vapors. There was a stronger dependence of the quantify of particles in e-vapors with the relative abundance of propylene glycol, glycerol and flavorings and to a lesser extend with nicotine content and applied voltage. These findings support the need to engage into practices and actions to discourage electronic cigarette vaping by new users, adults, adolescence and children; and regulate the manufacture and content of electronic cigarettes used as a smoking cessation tool.

## Materials and methods

### E-vapor generation

A large-volume fourth generation SMOK G150 TFV8 Baby Beast device was used to generate e-vapors. The device was composed of an in-built battery in a variable wattage (VW) mode of 6–225 W and a temperature range of 100–315 °C. Atomizers were used at 0.15 Ohm (SMOG V8 Baby-T8, stainless steel octuple coils, synthetic cotton, operating range 50–110 W). The atomizer was attached to 5 mL tanks filled with 3 mL of e-liquid at the beginning of every experiment. The puffing protocol included 2 secs puff and 15 secs wait time (puff volume: 20–30 mL) and was repeated sixteen (16) times to obtain multiple measurements. The puffing protocol was comparable to the modified puffing protocol (MPP; puff volume: 55 ml, puff duration: 4 s and puff interval: 30 s).^47^ The mouthpiece of the e-cig was attached to a 50 ml glass adapter connected to a continuous condensation particle counter (Model 3007, TSI Inc., Shoreview MN) and a real-time particle sizer (Model PC-2H, Sierra Madre, CA). The atomizer, tank, and glassware were replaced for each experiment to eliminate contamination. All glassware, including tanks, were cleaned with purified water acetone and dried prior to use. A blank sample was running for 60 s before each experiment. A new coil was used to reduce sample contamination.

### Particle number and size distribution monitoring

The condensation particle counter monitored particle number concentration (PNC, in part/cm^3^) with diameter from 10 nm up to 1 μm with a detection limit of 0.001 part/cm^3^ and 20% accuracy. The real-time 10-stage Quartz Crystal Microbalance (QCM) cascade impactor system was used to measure particle mass (in mg/m^3^) in ten stages with 50% cut-off aerodynamic diameter (d_a_) cut-off points of 14, 9.7, 4.2, 2.8, 1.4, 0.7, 0.45, 0.30, 0.16 and 0.10 μm at 2 L/min flow rate. The average sensitivity is 1.4 ng per Hz. For typical ambient conditions, the signal-to-noise (S/N) ratio was more than 20, sufficient to adequately measure particle mass without crystal overloading. The sensing crystals were cleaned between experiments with *n*-hexane and re-calibrated per the manufacturer’s protocol.

### Reference liquids and refill e-liquids

Sixteen reference solutions of propylene glycol (PG), glycerol (G), nicotine, and water at different ratios (Table S1 in Supplemental Information)^23^. Nicotine (3-(1-methyl-2-pyrrolidinyl)pyridine (N), C_10_H_14_N_2_, 99%) was purchased from Acros Chemicals (Pittsburg, PA). Propylene glycol (1,2-propanediol (PG), C_3_H_8_O_2_, 99.5%), glycerol (2,3-propanetriol (G), C_3_H_8_O_3_, 99 + %) and ultrapure water (HPLC grade) were obtained from Fisher Scientific (Waltham, MA). In addition, a total of twenty-two commercially available e-liquids at different nicotine concentrations (0%, 1.2%, and 2.4%) in the US market by a popular brand were used to assess the particles’ formation. Their chemical content has been previously characterized^23^. These e-liquids were purchased through the internet: blueberry, blueberry cobbler, cherry, Carolina bold, caramel café, gold leaf, menthol, mint chocolate, strawberry mint, tobacco, and vanilla.

### Data processing and analysis

PNC and mass concentrations were tested for normality using the Shapiro–Wilk test. The significance of difference in mean PNC between groups was examined with the parametric ANOVA tests at α = 0.05 followed by Tukey HSD. For each impactor stage, the normalized particle mass was computed as previously described^46^. The 1-min global max PNC and corresponding time (t_p,1_, s) were calculated for the reference and refill e-liquid solutions. In addition, PNC and corresponding times of subsequent local maxima (t_p,2_, s) and minima (troughs, hereafter, t_t_, s) if multiple peaks were identified were recorded. A PNC peak was designated when PNC increased for the two preceding measurements and decreasing for the two succeeding measurements. Similarly, a trough designation was given when PNC declined for two consecutive points followed by an increase for nest two measurements. In most cases, PNC change was higher than 20% (accuracy of the instrument). Multivariate linear regression analysis of PNC measured at t_p,1,_ t_p,2,_ and t_t_ against the chemical content of the parent refill e-liquid was done to determine their influence of e-vapor PNC (Eq. 1):

1 $$PNC = \sum\nolimits_{i = 1}^{n} {a_{i} } \cdot \left[ X \right]_{i} + a_{o}$$

where α_i_ and [X]_*I*_ were the regression coefficient and concentrations of the *i*-refill e-liquid components (PG, G, nicotine and non-exchangeable [*H*–C], [*H*–C–C =], [O–C–*H*], [*H*–C = C] + [O–C*H*–O], [*H*–Ar] and [*H*–C = O]) The intercept, α_0_, accounted for particles generated from other chemicals, including water. The abundance of water in e-liquids cannot be calculated because of the use of H_2_O/D_2_O solvent for the acquisition of ^1^H-NMR spectra. The coefficient of variation of the root mean square error, CV(RMSE), was used to evaluate the residuals between measured and predicted PNC. Regression analyses were done using SPSS (version 27, IBM Analytics).

## Supplementary Information

Supplementary Information.

## Data Availability

The datasets generated during during the current study are available from the corresponding author on reasonable request.

## References

[1] Etter J-F (2010). Electronic cigarettes: A survey of users. BMC Public Health.

[2] Hawkins KB, Johnson AC, Denzel M, Tercyak KP, Mays D (2017). Adolescents’s awareness and perceptions of e-cigarettes: Implications for intervention and tobacco regulation. Pediatrics.

[3] Pellegrino RM (2012). Electronic cigarettes: An evaluation of exposure to chemicals and fine particulate matter (PM). Ann. Ig..

[4] US Food and Drug Adminsitration, Lung illnesses associated with use of vaping products information for the public, FDA Actions, and Recommendations (2019).

[5] US Food and Drug Adminsitration, Statement on federal and state collaboration to investigate respiratory illnesses reported after use of e-cigarette products (2019).

[6] Goniewicz ML, Kuma T, Gawron M, Knysak J, Kosmider L (2013). Nicotine levels in electronic cigarettes. Nicotine Tob. Res..

[7] Margham J (2016). Chemical composition of aerosol from an e-cigarette: A quantitative comparison with cigarette smoke. Chem. Res. Toxicol..

[8] Bekki K, Uchiyama S, Ohta K, Inaba Y, Nakagome H, Kunugita N (2014). Carbonyl compounds generated from electronic cigarettes. Int. J. Environ. Res. Pub. Health.

[9] Kaur G, Pinkston R, McLemore B, Dorsey WC, Batra S (2018). Immunological and toxicological risk assessment of e-cigarettes. Eur. Respir. Rev..

[10] Belka M, Lizal F, Jedelsky J, Jicha M, Pospisil J (2017). Measurement of an electronic cigarette aerosol size distribution during a puff. EPJ Web Conf..

[11] Lampos S (2019). Real-time assessment of e-cigarettes and conventional cigarettes emissions: Aerosol size distributions, mass and number concentrations. Toxics.

[12] Ruprecht AA (2014). Comparison between particulate matter and ultrafine particle emission by electronic and normal cigarettes in real-life conditions. Tumori Journal.

[13] Scungio M, Stabile L, Buonanno G (2018). Measurements of electronic cigarette-generated particles for the evaluation of lung cancer risk of active and passive users. J. Aerosol Sci..

[14] Fuoco FC, Buonanno G, Stabile L, Vigo P (2014). Influential parameters on particle concentration and size distribution in the mainstream of e-cigarettes. Environ. Pollut..

[15] Zhao J, Nelson J, Dada O, Pyrgiotakis G, Kavouras IG, Demokritou P (2017). Assessing electronic cigarette emissions: Linking physico-chemical properties to product, liquid flavoring additives, operational voltage and user puffing patterns. Inhal. Toxicol..

[16] Floyd EL, Queimado L, Wang J, Regens JL, Johnson DL (2018). Electronic cigarette power affects count concentration and particle size distribution of vaping aerosol. PLoS ONE.

[17] Ingebrethsen BJ, Cole SK, Alderman SL (2012). Electronic cigarette aerosol particle size distribution measurements. Inhal. Toxicol..

[18] Zervas E, Litsiou E, Konstantopoulos K, Poulopoulos S, Katsaounou P (2018). Physical characterization of the aerosol of an electronic cigarette: impact of refill liquids. Inhal. Toxicol..

[19] Brown CJ, Cheng JM (2014). Electronic cigarettes: Product characterisation and design considerations. Tob. Control.

[20] Singh J, Luquet E, Smith DPT, Potgieter HJ, Ragazzon P (2016). Toxicological and analytical assessment of e-cigarette refill components on airway epithelia. Sci. Prog..

[21] Talih S (2015). Effects of user puff topography, device voltage, and liquid nicotine concentration on electronic cigarette nicotine yield: Measurements and model predictions. Nicotine Tob. Res..

[22] Hutzler C, Paschke M, Kruschinski S, Henkler F, Hahn J, Luch A (2014). Chemical hazards present in liquids and vapors of electronic cigarettes. Arch. Toxicol..

[23] Dada OM, Chalbot M-C, Kavouras IG (2020). Functional characterization of flavorings in electronic cigarette refill liquids by nuclear magnetic resonance spectroscopy. RSC Anal. Meth..

[24] Chacon-Madrid HJ, Presto AA, Donahue NM (2010). Functionalization vs. fragmentation: n-aldehyde oxidation mechanisms and secondary organic aerosol formation. Phys. Chem. Chem. Phys..

[25] Hamilton JF, Webb PJ, Lewis AC, Reviejo MM (2005). Quantifying small molecules in secondary organic aerosol formed during the photo-oxidation of toluene with hydroxyl radicals. Atmos. Environ..

[26] Ziemann PJ (2011). Effects of molecular structure on the chemistry of aerosol formation from the OH-radical-initiated oxidation of alkanes and alkenes. Int. Rev. Phys. Chem..

[27] Donahue NM, Trump ER, Pierce JR, Riipinen I (2011). Theoretical constraints on pure vapor-pressure driven condensation of organics to ultrafine particles. Geophys. Res. Lett..

[28] Kavouras IG, Stephanou EG (2002). Direct evidence of atmospheric secondary organic aerosol formation in forest atmosphere through heteromolecular nucleation. Environ. Sci. Technol.

[29] Stull DR (1947). Correction - Vapor pressure of pure substances. Ind. Engin. Chem..

[30] Young HD, Nelson OA (1929). Vapor pressures of fumigants IV–vapor vressure of nicotine. Ind. Engin. Chem..

[31] Salma I (2011). Production, growth and properties of ultrafine atmospheric aerosol particles in an urban environment. Atmos. Chem. Phys..

[32] Boublík T, Fried V, Hála E (1973). The Vapour Pressures of Pure Substances-Selected Values of the Temperature Dependence of the Vapour Pressures of Some Pure Substances in the Normal and Low Pressure Region.

[33] Kavouras IG, Mihalopoulos N, Stephanou EG (1998). Formation of atmospheric particles from organic acids produced by forests. Nature.

[34] Zhang R (2004). Atmospheric new particle formation enhanced by organic acids. Science.

[35] Aschmann SM, Atkinson R (1998). Kinetics of the gas-phase reactions of the oh radical with selected glycol ethers, glycols, and alcohols. Int. J. Chem. Kin..

[36] Borduas N, Murphy JG, Wang C, da Silva G, Abbatt JPD (2016). Gas phase oxidation of nicotine by OH radicals: Kinetics, mechanisms, and formation of HNCO. Environ. Sci. Technol. Lett..

[37] Stein YS, Antal MJ, Jones M (1983). A study of the gas-phase pyrolysis of glycerol. J. Anal. Appl. Pyrol..

[38] Goniewicz ML (2014). Levels of selected carcinogens and toxicants in vapour from electronic cigarettes. Tob. Control.

[39] Saffari A (2014). Particulate metals and organic compounds from electronic and tobacco-containing cigarettes: Comparison of emission rates and secondhand exposure. Environ. Sci. Process Impacts.

[40] Atkinson R (1986). Kinetics and mechanisms of the gas-phase reactions of the hydroxyl radical with organic compounds under atmospheric conditions. Chem. Rev..

[41] US Environmental Protection Agency (EPA). CompTox Chemistry Dashboard. (FDA, 2019).

[42] Tolocka MP, Jang M, Ginter JM, Cox FJ, Kamens RM, Johnston MV (2004). Formation of oligomers in secondary organic aerosol. Environ. Sci. Technol..

[43] Bahl V, Lin S, Xu N, Davis B, Wang YH, Talbot P (2012). Comparison of electronic cigarette refill fluid cytotoxicity using embryonic and adult models. Reprod. Toxicol..

[44] Farsalinos KE (2013). Comparison of the cytotoxic potential of cigarette smoke and electronic cigarette vapour extract on cultured myocardial cells. Int. J Environ. Res. Public Health.

[45] Schober W (2014). Use of electronic cigarettes (e-cigarettes) impairs indoor air quality and increases FeNO levels of e-cigarette consumers. Int. J. Hyg. Environ. Health.

[46] Kavouras IG, Stephanou EG (2002). Gas/particle partitioning and size distribution of primary and secondary carbonaceous aerosol in public buildings. Indoor Air.

[47] Farsalinos KE, Romagna G, Tsiapras D, Kyrzopoulos S, Voudris A (2013). Evaluation of electronic cigarette use (Vaping) topography and estimation of liquid consumption: Implications for research protocol standards definition and for public health authorities regulation. Int. J. Environ. Res. Public Health.

