# Supplementary material for: Evidence for the coupling of refill liquids content and new particle formation in electronic cigarette vapors
Source: Sci Rep. 2022 Nov 3;12:18571. doi: 10.1038/s41598-022-21798-w (PMC9633786; doi:10.1038/s41598-022-21798-w)
Supplement: Supplementary file 1 — Supplementary Information. [file 41598_2022_21798_MOESM1_ESM.docx]

**Evidence for the coupling of refill liquids content and new particle formation in electronic cigarette vapors**

Oluwabunmi Dada^1,3^, Karina Castillo^2^, Miranda Hogan^2^, Marie-Cecile G. Chalbot^1,4^ & Ilias G. Kavouras^1,2,🖂^

^1^ Department of Environmental Health Sciences, University of Alabama at Birmingham, Birmingham, AL 35219, USA

^2^ Department of Environmental, Occupational and Geospatial Health Sciences, CUNY Graduate School of Public Health and Health Policy, New York, NY 10025, USA

^3^ Department of Occupational Safety, Murray State University, Murray, KY 42071, USA

^4^ Department of Biological Sciences, CUNY College of Technology, Brooklyn, NY 11201, USA

^🖂^ e-mail: [ilias.kavouras@sph.cuny.edu](mailto:ilias.kavouras@sph.cuny.edu)

**Supplemental Information**

| **PG:G:**  **Water:nicotine** | **PNC (part/cm^3^)** | **t_p,1_ (s)** | **Δt (s)** |
| --- | --- | --- | --- |
| 100:0:0:0 | 48,411 ± 10,901 (315,154) | 3.3 ± 1.8 | 4.1 ± 0.1 |
| 0:100:0:0 | 68,768 ± 10,798 (328,542) | 4.3 ± 1.3 | 3.7 ± 0.2 |
| 0:0:100:0 | 3,092 ± 443 (18,440) | 2.7 ± 1.4 | 4.8 ± 0.1 |
| 0:0:0:100 | 52,288 ± 8,524 (209,158) | 2.7 ± 0.8 | 4.6 ± 0.2 |
| 50:50:0:0 | 113,429 ± 15,119 (318,626) | 3.8 ± 1.0 | 5.0 ± 0.5 |
| 60:40:0:0 | 147,972 ± 18,625 (318,506) | 3.5 ± 1.4 | 3.7 ± 0.2 |
| 70:30:0:0 | 87,424 ± 17,261 (318,046) | 3.3 ± 3.0 | n.d. |
| 80:20:0:0 | 129,018 ± 29,814 (348,166) | 3.8 ± 0.8 | n.d. |
| 45:45:10:0 | 110,491 ± 17,016 (315,720) | 3.5 ± 0.5 | n.d. |
| 45:45:9.4:0.6 | 124,869 ± 14,111 (344,221) | 3.4 ± 1.3 | 4.7 ± 0.3 |
| 45:45:9:1 | 131,007 ± 21,378 (337,433) | 4.1 ± 0.7 | n.d. |
| 45:45:7.5:2.5 | 99,132 ± 16,441 (334,108) | 3.8 ± 1.5 | n.d. |
| 60:30:10:0 | 73,784 ± 13,059 (352,754) | 3.2 ± 1.4 | 3.7 ± 0.2 |
| 60:30:9.4:0.6 | 147,972 ± 18,625 (318,506) | 3.5 ± 1.4 | 3.7 ± 0.2 |
| 60:30:9:1 | 106,369 ± 16,709 (317,947) | 3.9 ± 1.0 | 5.0 ± 0.1 |
| 60:30:7.5:2.5 | 74,921 ± 16,603 (337,191) | 3.3 ± 1.2 | 5.5 ± 0.1 |

**Table S1.** Summary of PNC concentrations and events in e-vapors from reference solutions. ± standard error of the mean (n=16), maximum PNC in parentheses. PNC particle number concentration, part/cm^3^ particles per cubic centimeter, t_p,1_ time of the first peak PNC, s seconds, Δt time between two consecutive PNC, PG, propylene glycol, G glycerol, n.d. not detected.

| **Flavor** | **PG:G** | **Nicotine (mg/ml)** | **FMR** | **PNC (part/cm^3^)** | **t_p,1_ (s)** | **Δt (s)** |
| --- | --- | --- | --- | --- | --- | --- |
| Blueberry | 60:40 | 1.2 | 0.049 | 98138 ± 15937 (282348) | 2.7 ± 1.1 | 4.3 ± 0.2 |
| Blueberry | 60:41 | 2.4 | 0.062 | 76994 ± 15538 (279356) | 3.6 ± 1.1 | 4.0 ± 0.2 |
| Blueberry cobbler | 35:65 | 1.2 | 0.082 | 108613 ± 13798 (293068) | 3.3 ± 0.9 | 5.7 ± 0.3 |
| Blueberry cobbler | 40:60 | 2.4 | 0.066 | 110623 ± 13705 (319968) | 2.9 ± 1.1 | 4.3 ± 0.2 |
| Cherry | 30:70 | 1.2 | 0.031 | 166178 ± 15930 (368785) | 2.4 ± 0.8 | 4.6 ± 0.2 |
| Blueberry cobbler | 30:70 | 2.4 | 0.033 | 149690 ± 13956 (355790) | 2.5 ± 0.8 | 5.5 ± 0.1 |
| Carolina Bold | 50:50 | 1.2 | 0.018 | 95479 ± 15678 (308826) | 2.1 ± 0.8 | 5.4 ± 0.4 |
| Carolina Bold | 45:55 | 2.4 | 0.013 | 102537 ± 12104 (343976) | 2.3 ± 0.9 | 5.3 ± 0.2 |
| Caramel Café | 40:60 | 1.2 | 0.049 | 90486 ± 17136 (333085) | 3.5 ± 0.8 | n.d. |
| Caramel Café | 40:60 | 2.4 | 0.065 | 102792 ± 15358 (302824) | 3.5 ± 0.9 | 5.0 ± 0.2 |
| Gold Leaf | 50:50 | 0 | 0.027 | 118792 ± 11906 (311151) | 2.4 ± 1.0 | 5.1 ± 0.2 |
| Menthol | 50:50 | 0 | 0.03 | 103879 ± 11053 (289063) | 2.5 ± 1.3 | 5.4 ± 0.2 |
| Menthol | 30:70 | 2.4 | 0.045 | 107806 ± 14730 (296143) | 2.7 ± 0.8 | 4.3 ± 0.1 |
| Mint Chocolate | 65:35 | 1.2 | 0.032 | 87921 ± 10364 (335137) | 1.7 ± 2.5 | 5.4 ± 0.2 |
| Mint Chocolate | 50:50 | 2.4 | 0.026 | 82870 ± 10678 (324781) | 2.5 ± 0.9 | 6.0 ± 0.2 |
| Strawberry Mint | 40:60 | 1.2 | 0.047 | 100622 ± 12541 (345304) | 2.0 ± 0.8 | 4.7 ± 0.3 |
| Strawberry Mint | 40:60 | 2.4 | 0.034 | 88487 ± 13583 (299083) | 2.6 ± 1.2 | 6.5 ± 0.4 |
| Tobacco | 30:70 | 1.2 | 0.022 | 83471 ± 12278 (290495) | 2.2 ± 0.8 | 5.2 ± 0.2 |
| Tobacco | 40:60 | 2.4 | 0.015 | 115379 ± 13032 (326783) | 2.2 ± 0.8 | 5.2 ± 0.2 |
| Vanilla | 40:60 | 0 | 0.012 | 64351 ± 12214 (273166) | 3.6 ± 1.4 | 3.0 ± 0.1 |
| Vanilla | 40:60 | 1.2 | 0.022 | 122748 ± 12948 (337514) | 3.2 ± 1.0 | 4.7 ± 0.1 |
| Vanilla | 40:60 | 2.4 | 0.035 | 95700 ± 14212 (310087) | 3.0 ± 1.1 | 4.3 ± 0.3 |

**Table S2.** Summary of PNC concentrations and events in e-vapors from commercial refill e-liquids. ± standard error of the mean (n=16), maximum PNC in parentheses. PNC particle number concentration, part/cm^3^ particles per cubic centimeter, t_p,1_ time of the first peak PNC, s seconds, Δt time between two consecutive PNC, PG, propylene glycol, G glycerol, FMR flavorings molar ratio characterized using proton nuclear magnetic resonance (^1^H-NMR) spectroscopy^23^,n.d. not detected.

| **Component** | **Reference solutions** | | | **E-liquids** | | |
| --- | --- | --- | --- | --- | --- | --- |
|  | **PNC at t_p,1_** | **PNC at t_t_** | **PNC at t_p,2_** | **PNC at t_p,1_** | **PNC at t_t_** | **PNC at t_p,2_** |
| PG | 0.55 | 0.27 | 0.19 | -0.24 | -0.40 | -0.46 |
| G | 0.36 | 0.31 | 0.34 | 0.47 | 0.29 | 0.20 |
| Water | -0.66 | -0.40 | -0.40 | N.d. | N.d. | N.d. |
| Nicotine | -0.33 | -0.24 | -0.16 | 0.19 | 0.12 | 0.39 |
| [H-C] | - | - | - | -0.15 | -0.16 | 0.04 |
| [H-C-C=] | - | - | - | 0.09 | 0.36 | 0.56 |
| [H-C-O] | - | - | - | -0.22 | 0.03 | 0.22 |
| [H-C=C] and [O-CH-O] | - | - | - | -0.21 | 0.14 | 0.36 |
| [H-Ar] | - | - | - | -0.15 | 0.24 | 0.58 |
| [H-C-O] | - | - | - | -0.18 | 0.21 | 0.53 |

**Table S3.** Summary of correlations of PNC concentrations at t_p,1_, t_t_, and t_p,2_ timepoints in e-vapors from reference solutions and commercial refill e-liquids^a^. ± standard error of the mean (n=16), maximum PNC in parentheses. PNC particle number concentration, part/cm^3^ particles per cubic centimeter, t_p,1_ time of the first peak PNC, s seconds, Δt time between two consecutive PNC, PG, propylene glycol, G glycerol, [H-C] saturated aliphatic, [H-C-C=] unsaturated aliphatic, [H-C-O] saturated oxygenated, [H-C=C] vinylic, [O-CH-O] acetalic, [H-Ar] aromatic, [H-C=O] carbonyl non-exchangeable organic hydrogen. ^a^ refill e-liquid composition was characterized using proton nuclear magnetic resonance (^1^H-NMR) spectroscopy^23^.

**Computations of molar and molecular concentration at 25^o^C and 198.75^o^C**

***Molar concentration (n/V; mol/lit)***

Ideal gas law: $n/V=(P)/(R\cdot T)$

P = compound’s vapor pressure (atm) (a factor of 1/760 was used to convert mmHg to atm)

R = ideal gas law constant (0.082 $(atm\cdot lit))/((mol\cdot{}^{o}K)$)

T = temperature (^o^K)

***Molecular concentration (N/V; in molecules/lit***

$$N/V=n/V\cdot6.023\cdot{10}^{23} molecules/mol$$

***Antoine equation***

$$logP=A-(B/(T+C))$$

For T = 471.94 ^o^K (198.75 ^o^C)

|  | **Nicotine**  **(407.0 - 519.4 ^o^K)** | **Propylene glycol**  **(318.7 - 461.4 ^o^K)** | **Glycerol**  **(456.40 - 533.6 ^o^K)** |
| --- | --- | --- | --- |
| A | 3.60721 | 6.07936 | 3.93737 |
| B | 1433.766 | 2692.187 | 1411.531 |
| C | -121.387 | -17.94 | -200.566 |

**Table S4.** Antoine equation coefficients and computed vapor pressure based on data compiled at NIST Chemistry WebBook, SRD 69.

| **Compound** | **V_p_ at 25^o^C** | **V_p_ at 198.75^o^C** |
| --- | --- | --- |
| Nicotine | 0.038 mmHg | 0.33 atm |
| Propylene glycol | 0.129 mmHg | 1.39 atm |
| Glycerol | 1.68 10^-4^ mmHg | 0.05 atm |
| Vanillin | 1.18 10^-4^ mmHg | - |
| Benzaldehyde | 1.27 mmHg | - |
| Menthol | 0.11 mmHg | - |

**Table S5.** Vapor pressures of e-vapors components at 25^o^C and 198.75^o^C. V_p_ vapor pressure.
